# Successful Outcomes with Oral Fluoroquinolones Combined with Rifampicin in the Treatment of *Mycobacterium ulcerans:* An Observational Cohort Study

**DOI:** 10.1371/journal.pntd.0001473

**Published:** 2012-01-17

**Authors:** Daniel P. O'Brien, Anthony McDonald, Peter Callan, Mike Robson, N. Deborah Friedman, Andrew Hughes, Ian Holten, Aaron Walton, Eugene Athan

**Affiliations:** 1 Department of Infectious Diseases, Barwon Health, Geelong, Australia; 2 Department of Medicine and Infectious Diseases, Royal Melbourne Hospital, University of Melbourne, Melbourne, Australia; 3 Department of Plastic Surgery, Barwon Health, Geelong, Australia; 4 Department of Pathology, Pathcare, Geelong, Australia; Fondation Raoul Follereau, France

## Abstract

**Background:**

The World Health Organization currently recommends combined streptomycin and rifampicin antibiotic treatment as first-line therapy for *Mycobacterium ulcerans* infections. Alternatives are needed when these are not tolerated or accepted by patients, contraindicated, or neither accessible nor affordable. Despite *in vitro* effectiveness, clinical evidence for fluoroquinolone antibiotic use against *Mycobacterium ulcerans* is lacking. We describe outcomes and tolerability of fluoroquinolone-containing antibiotic regimens for *Mycobacterium ulcerans* in south-eastern Australia.

**Methodology/Principal Findings:**

Analysis was performed of prospectively collected data including all primary *Mycobacterium ulcerans* infections treated at Barwon Health between 1998 and 2010. Medical treatment involved antibiotic use for more than 7 days; surgical treatment involved surgical excision of a lesion. Treatment success was defined as complete lesion healing without recurrence at 12 months follow-up. A complication was defined as an adverse event attributed to an antibiotic that required its cessation. A total of 133 patients with 137 lesions were studied. Median age was 62 years (range 3–94 years). 47 (34%) had surgical treatment alone, and 90 (66%) had combined surgical and medical treatment. Rifampicin and ciprofloxacin comprised 61% and rifampicin and clarithromycin 23% of first-line antibiotic regimens. 13/47 (30%) treated with surgery alone failed treatment compared to 0/90 (0%) of those treated with combination medical and surgical treatment (p<0.0001). There was no difference in treatment success rate for antibiotic combinations containing a fluoroquinolone (61/61 cases; 100%) compared with those not containing a fluoroquinolone (29/29 cases; 100%). Complication rates were similar between ciprofloxacin and rifampicin (31%) and rifampicin and clarithromycin (33%) regimens (OR 0.89, 95% CI 0.27–2.99). Paradoxical reactions during treatment were observed in 8 (9%) of antibiotic treated cases.

**Conclusions:**

Antibiotics combined with surgery may significantly increase treatment success for *Mycobacterium ulcerans* infections, and fluoroquinolone combined with rifampicin-containing antibiotic regimens can provide an effective and safe oral treatment option.

## Author Summary

Buruli ulcer is a necrotizing infection of skin and subcutaneous tissue caused by *Mycobacterium ulcerans* and is the third most common mycobacterial disease worldwide (after tuberculosis and leprosy). In recent years its treatment has radically changed, evolving from a predominantly surgically to a predominantly medically treated disease. The World Health Organization now recommends combined streptomycin and rifampicin antibiotic treatment as first-line therapy for *Mycobacterium ulcerans* infections. However, alternatives are needed where recommended antibiotics are not tolerated or accepted by patients, contraindicated, or not accessible nor affordable. This study describes the use of antibiotics, including oral fluoroquinolones, in the treatment of *Mycobacterium ulcerans* in south-eastern Australia. It demonstrates that antibiotics combined with surgery are highly effective in the treatment of *Mycobacterium ulcerans*. In addition, oral fluoroquinolone-containing antibiotic combinations are shown to be as effective and well tolerated as other recommended antibiotic combinations. Fluoroquinolone antibiotics therefore offer the potential to provide an alternative oral antibiotic to be combined with rifampicin for *Mycobacterium ulcerans* treatment, allowing more accessible and acceptable, less toxic, and less expensive treatment regimens to be available, especially in resource-limited settings where the disease burden is greatest.

## Introduction

In recent years the treatment of *Mycobacterium ulcerans (M. ulcerans)* has radically changed, evolving from a predominantly surgically [1], [2] to a predominantly medically treated disease [3]. This resulted from clinical experience supported by scientific data showing superior outcomes when antibiotics were used alone [4], [5], or combined with surgery [6], [7]. It is also supported by in vitro data [8]–[10] of antibiotic effectiveness against *M. ulcerans*. The World Health Organization now recommends combined streptomycin and rifampicin antibiotic treatment as first-line therapy for *M. ulcerans* infections, with surgery reserved mainly to remove necrotic tissue, cover skin defects, and correct deformities [11].

The Bellarine Peninsula in south-eastern Australia has been experiencing an epidemic of *M. ulcerans* since 1998. It affects local residents, but also visitors from outside the endemic region, with cases in those living locally managed at the local referral health service, Barwon Health. In our region, despite recommendations at the time against their use, adjunctive antibiotic treatment of *M. ulcerans* was initiated from 1998 in response to severe disease causing significant morbidity and requiring reconstructive surgery, and disease recurrences despite surgery. Fluoroquinolones (FQ) were introduced into antibiotic regimens in 2003 [6], [12] in response to perceived treatment failures and excess toxicity with other antibiotics, as well as their potential advantages in treating *M. ulcerans*; documented in vitro evidence of activity [9], [10], [13]–[15], good bioavailability [16], and excellent bone and tissue penetration [17]. Since their introduction they have commonly been employed in the antibiotic regimens used at Barwon Health.

FQ antibiotics offer the possibility of completely oral antibiotic regimens when combined with another active oral antibiotic, usually rifampicin. This can be especially useful where other recommended antibiotics are not tolerated, accepted, accessible, or affordable. However, although there is evidence of good activity against *M. ulcerans* in the laboratory [9]–[10], [13], in mouse footpad models [8], [14], [18], and small numbers of clinical cases [6], [12], [19]–[21], clinical evidence of FQ efficacy is lacking. Therefore we undertook a study during the current epidemic in the Bellarine Peninsula to describe the use of FQ antibiotics in *M. ulcerans* treatment and to compare their outcomes and tolerability with other antibiotics used.

## Methods

### Participants

Analysis was performed using prospectively collected data from an electronic database containing information on all cases of *M. ulcerans* infection treated at Barwon Health between 1^st^ March 1998 and 31^st^ May 2010. Data collected includes epidemiological details, diagnostic tests, clinical features, treatment, and outcomes. Only first lesions on initial presentation were analyzed to avoid potential confounders when analyzing recurrent cases. Data was analyzed anonymously.

### Definitions

A case of *M. ulcerans* was defined as the presence of a lesion clinically suggestive of *M. ulcerans* plus any of (1) a culture of *M. ulcerans* from the lesion, (2) a positive PCR from a swab or biopsy of the lesion [22], or (3) histopathology of an excised lesion showing a necrotic granulomatous ulcer with the presence of acid-fast bacilli consistent with acute *M. ulcerans* [23].

Surgical treatment was defined as the surgical excision of a lesion. Due to the paucity of cases managed without surgery, only those undergoing surgery were included. Major surgery involved the use of a split thickness skin graft or a vascularized tissue flap to close the tissue defect, whereas minor surgery involved excision plus primary closure of the defect. A positive margin was defined on histology as granulomatous inflammation or necrotic tissue extending to the margins of the excised lesion.

Medical treatment was defined as the use of antibiotics for more than 7 days, and first-line regimens were the initial antibiotic regimens used. A complication was an adverse event attributed to an antibiotic that required the cessation of that medication. Drug dosages for adults included ciprofloxacin 500 mg twice daily, moxifloxacin 400 mg daily, rifampicin 10 mg/kg/day (up to a maximum of 600 mg daily), clarithromycin 500 mg twice daily, and amikacin 15 mg/kg/day.

Treatment success was defined as complete healing of the lesion without recurrence 12 months after treatment commencement. Treatment failure was defined as those who had a recurrence with in at least 12 months of follow-up. Recurrence was defined as a new lesion appearing either in the wound, locally, or in another part of the body after the surgical excision of the initial lesion that met the case definition for *M. ulcerans*. Paradoxical reactions were not considered a recurrence and were defined as: initial clinical improvement followed by the clinical deterioration of the treated lesion, or the appearance of a new lesion, either locally or in a distant body site, that on histopathology showed evidence of an intense immunological reaction consistent with an immune-mediated paradoxical reaction [19].

There was no standardized treatment protocol for *M. ulcerans* followed in Barwon Health during the study period. The role of surgery and the use of antibiotics were determined by individual specialist practitioners involved in *M. ulcerans* treatment. Patients were followed up according to routine clinical practice and observed antibiotic complications recorded in clinical notes when they occurred.

### Ethics

This study was approved by the Barwon Health Research and Ethics Committee.

### Statistics

Data were collected and analysed with Epi-Info 6 (Centers for Disease Control, Atlanta). Statistical significance was determined using the 2-tailed Fisher exact test for 2×2 tables for each of the categorical values. A non-parametric cumulative failure graph using the Kaplan-Meier method and the endpoint of antibiotic cessation was plotted using the statistics package Minitab (version 15) to model the proportion of antibiotics ceased over time due to complications.

## Results

One hundred and forty seven patients with *M. ulcerans* were diagnosed and treated at Barwon Health over the study period 1^st^ March 1998 to 31^st^ May 2010. Fourteen were excluded from further analysis: 1 had no surgery, 2 died before the completion of follow-up (1 from a cerebrovascular accident 52 days and 1 of sepsis secondary to the *M. ulcerans* lesion 5 days post treatment commencement), 1 was lost to follow-up 85 days post treatment commencement, and for 10 treatment and follow-up was ongoing. Therefore a total of 133 patients with 137 lesions (4 patients had 2 lesions at initial presentation) were included in the analysis.

Median age of patients was 62 years (range 3–94 years); 7 (5%) were <15 years. Sixty-seven (50%) were male. Associated co-morbidities included diabetes mellitus (11), malignancy (5), connective tissue disease (4), and immunosuppressive treatment (4).

For 122 cases where the clinical type of lesion was recorded, 106 (87%) were ulcers, 9 (7%) were nodules, and 7 (6%) were oedematous lesions. Diagnosis was made by PCR in 116 (87%), positive culture in 22 (17%), and consistent histopathology in 54 (41%) cases. Eighteen of 24 (75%) PCR positive cases where culture was performed were culture positive, but no cases were culture positive and PCR negative. One case was PCR negative but positive on histopathology.

**Figure 1:**
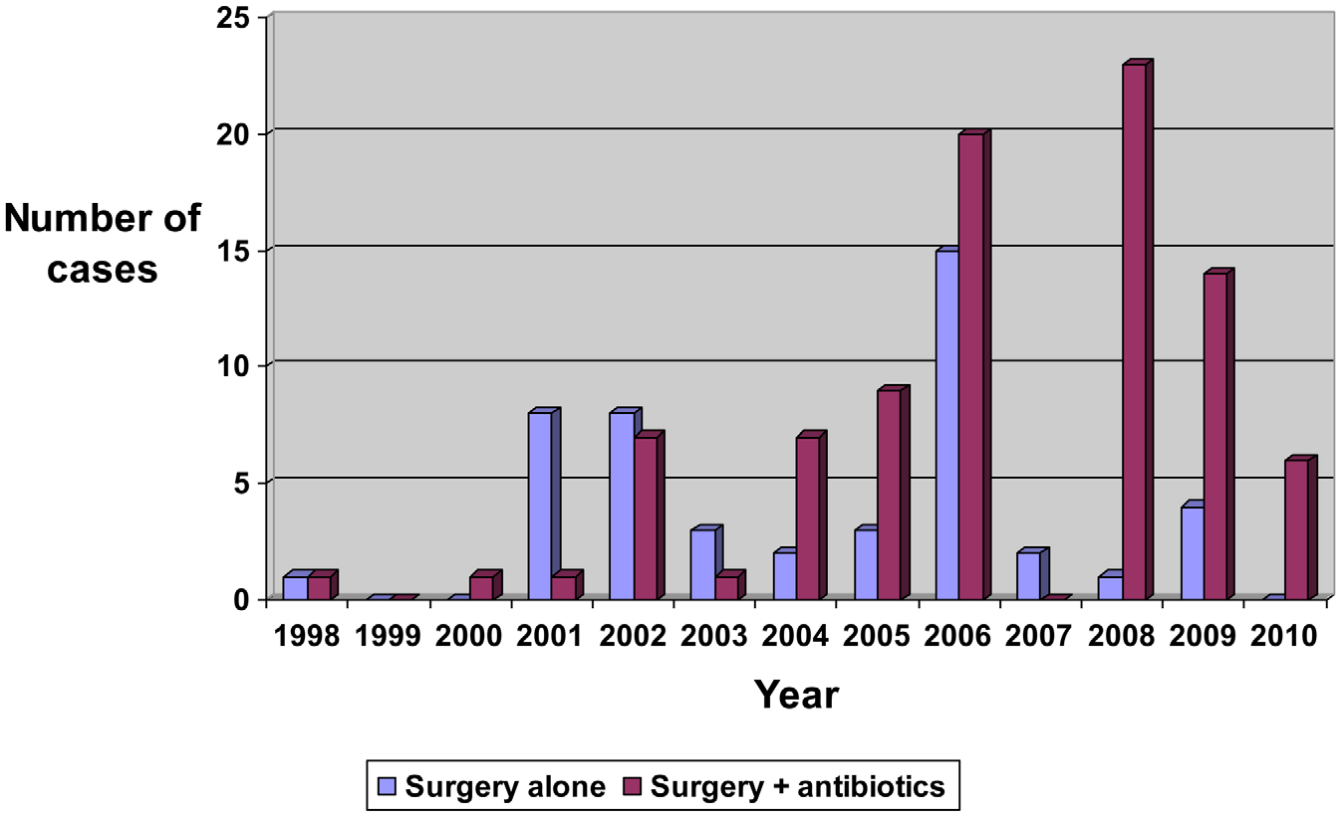
Type of treatment for initial *M. ulcerans* lesions.

Forty-seven (34%) lesions were treated with surgical excision alone, and 90 (66%) had surgical excision and adjunctive antibiotic therapy. To close the skin defect after excision of the lesion, 65 (47%) required a split thickness skin graft and 16 (12%) required a vascularized tissue flap. The proportion of cases receiving antibiotics significantly increased pre 2005 compared to post 2005, rising from 45% to 74% [<2005 18/40, ≥2005 72/97, OR 3.52 (1.52–8.20)] (Figure 1).

The most common initial antibiotic regimens were rifampicin and ciprofloxacin (61%) and rifampicin and clarithromycin (23%). Four patients received ciprofloxacin and clarithromycin, and 2 patients received rifampicin and moxifloxacin regimens (Table 1). FQ antibiotics were used in 3 of 5 children aged <15 years who received antibiotics. Antibiotics were given for a median duration of 76 days (range 12–155 days); 21 (23%) between 12–30 days, 18 (20%) 31–60 days, 30 (33%) 61–90 days, 14 (16%) 91–120 days, and 7 (8%) 121–155 days. FQ antibiotics were not used in this study until 2004, but from then 82% of regimens were FQ containing.

**Table 1:** First-line antibiotic regimens and complication rates.

Antibiotic regimen	First-line (n = 90)	Complication
Rifampicin and ciprofloxacin	55 (61%)	17 (31%)
Rifampicin and clarithromycin	21 (23%)	7 (33%)
Rifampicin, clarithromycin, and ethambutol	5 (4%)	2 (40%)
Ciprofloxacin and clarithromycin	4 (4%)	0 (0%)
Rifampicin and moxifloxacin	2 (2%)	1(50%)
Clarithromycin and ethambutol	1 (1%)	0(0%)
Rifampicin, ethambutol, and amikacin	1 (1%)	1(100%)
Clarithromycin	1 (1%)	0 (0%)

Fourteen of 47 (30%) of those treated with surgery alone failed treatment compared to 0/90 (0%) of those treated with a combination of medical and surgical treatment (p<0.0001). The risk of treatment failure increased significantly with no antibiotics compared to those treated with antibiotics for major surgery (p<0.0001), minor surgery (p = 0.01), positive margins (p<0.0001), and negative margins (p = 0.05) (Table 2). If minor surgery and negative margins were present, 1/22 (5%) failed treatment. If only regimens containing an FQ (n = 64) are compared with surgery alone, the risk of treatment failure remained significantly increased when no antibiotics were used overall (p<0.0001) and for major surgery (p<0.0001) and positive margins (p<0.0001) (Table 3). There was no difference in treatment success rate for antibiotic combinations containing an FQ (61/61 cases; 100%) compared with those not containing an FQ (29/29 cases; 100%). Treatment success rates with antibiotics were also similar pre-2004 when no FQs were used (11/11; 100%) compared with post 2004 (79/79; 100%). All four cases treated with ciprofloxacin and clarithromycin combined with surgery experienced treatment success.

**Table 2:** Proportion of cases with treatment success for all initial *M. ulcerans* lesions.

	Total antibiotics+no antibiotics	Received antibiotics	No antibiotics	P-value
All first episodes	123/137 (90%)	90/90 (100%)	33/47 (70%)	p<0.0001
Minor surgery	53/59 (90%)	30/30 (100%)	23/29 (79%)	p = 0.01
Major surgery	70/78(90%)	60/60 (100%)	10/18 (56%)	p<0.0001
Negative margins	64/68 (94%)	35/35 (100%)	29/33 (88%)	p = 0.05
Positive margins	59/69 (86%)	55/55 (100%)	4/14 (29%)	p<0.0001

**Table 3:** Proportion of cases with treatment success for initial *M. ulcerans* lesions using fluoroquinolone-containing regimens.

	Total antibiotics+no antibiotics	Received antibiotics	No antibiotics	P-value
All first episodes	97/111 (87%)	64/64 (100%)	33/47 (70%)	p<0.0001
Minor surgery	43/49 (88%)	20/20 (100%)	23/29 (79%)	p = 0.07
Major surgery	54/62 (87%)	44/44 (100%)	10/18 (56%)	p<0.0001
Negative margins	51/55 (93%)	22/22 (100%)	29/33 (88%)	p = 0.14
Positive margins	44/54 (81%)	40/40 (100%)	4/14 (29%)	p<0.0001

For those failing treatment, the recurrences occurred a median 90 days post surgery (range 14–300 days). In 9 (64%) patients this was local and in 6 (43%) patients it was distant (1 had both). Paradoxical reactions occurred in 8/90 (9%) of cases given antibiotics after a median duration of 48 days (range 14–85 days).

Fifty-eight (64%) patients had antibiotics prior to surgery for a median duration of 8 days (range 1–36 days). Of these, mycobacterial cultures were performed on 28 excised specimens. Cultures were positive for *M. ulcerans* in 11/20 (55%) of those who received ≤14 days of antibiotics prior to surgery, and in 1/8 (12.5%) of those who received >14 days of antibiotics prior to surgery (Table 4). All cases with positive cultures were associated with successful outcomes after a median antibiotic duration of 87 days (range 30–155 days).

**Table 4:** Mycobacterial culture results for specimens after >14 days of antibiotics prior to surgical excision.

Antibiotic regimen	Antibiotic duration prior to culture (days)	Mycobacterial Culture	Total antibiotic duration (days)	Outcome
RCCp	18	Negative	23	Treatment success
RCp	20	Negative	132	Treatment success
RCp	33	Negative	90	Treatment success
RCp	36	Positive	119	Treatment success
RCp	41	Negative	88	Treatment success
RC	46	Negative	62	Treatment success
RCEA	65	Negative	65	Treatment success
RCCp	86	Negative	137	Treatment success

R = rifampicin, C = clarithromycin, Cp = ciprofloxacin, E = ethambutol, A = amikacin.

Rifampicin was associated with a complication in 19/85 (22%) cases occurring at a median 27 days (range 6–94 days) and involved gastrointestinal intolerance in 15, hepatitis 4, rash 3, and hypoglycemia in 1 case. Ciprofloxacin was associated with a complication in 13/63 (21%) cases occurring at a median 24 days (range 6–90 days) and involved gastrointestinal intolerance in 10, joint or tendon effects in 3, rash in 2, and hallucinations in 1 case. Clarithromycin was associated with a complication in 10/38 (26%) cases occurring at a median 25 days (range 2–60 days) and involved gastrointestinal intolerance in 9, hepatitis in 1, and palpitations in 1 case.

**Figure 2:**
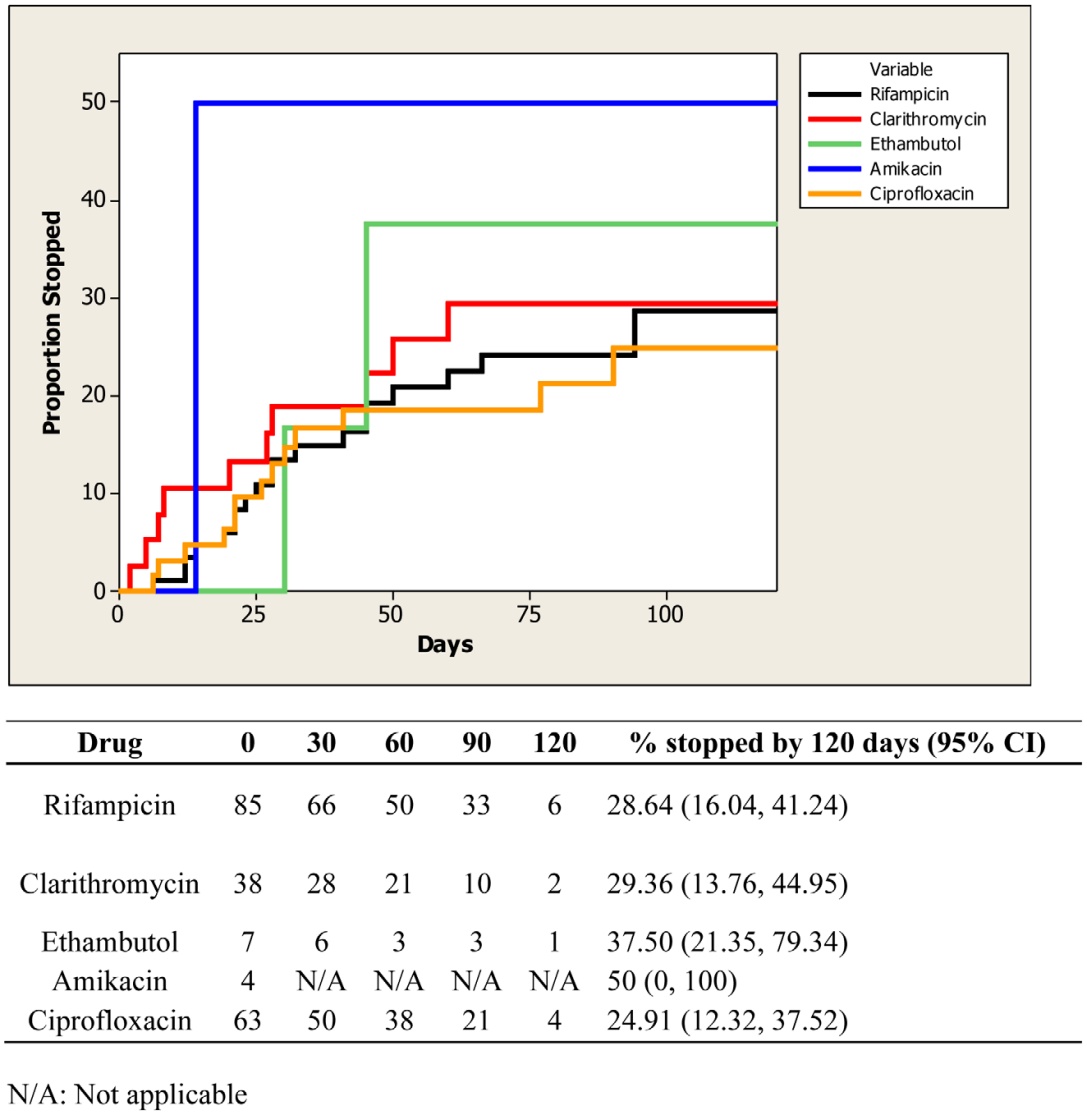
Proportion of individual antibiotics ceased over time due to complications.

By 120 days on treatment, the proportion of cases in which rifampicin [28.6% (95% CI 16.0, 41.2)], clarithromycin [29.4% (95% CI 13.8, 45.0)], and ciprofloxacin [24.9% (95% CI 12.3, 37.5)] were ceased were similar (Figure 2), and complication rates were similar between ciprofloxacin and rifampicin 17/55 (31%) and rifampicin and clarithromycin 7/21 (33%) regimens (OR 0.89, 95% CI 0.27–2.99).

## Discussion

Our study demonstrates that, combined with surgical excision of *M. ulcerans* lesions, antibiotics appear highly effective at preventing disease recurrences; we describe a reduction of recurrence rates from more than one quarter of cases with surgery alone to none if antibiotics are used. This includes 64 cases treated with FQ-containing regimens (the majority involving ciprofloxacin) which show similar efficacy to non-FQ containing regimens. Recent studies have provided strong evidence that non-FQ-containing antibiotic regimens were effective in *M. ulcerans* treatment; 8-week combinations of rifampicin and streptomycin cured 96% of cases in a randomized controlled trial in Africa [5], and 8 weeks of rifampicin and clarithromycin were 100% effective in an uncontrolled trial in Benin [4]. Although we have previously published small numbers of *M. ulcerans* cases treated with FQ-containing regimens [6], [12], [19], this is the first study large enough to provide significant evidence of the clinical effectiveness of FQ antibiotics combined mainly with rifampicin in *M. ulcerans* treatment.

Ciprofloxacin has been shown to have good in vitro activity against *M. ulcerans* with minimal inhibitory concentrations (MIC) of between 0.5 and 1 mg/l in two published studies [9], [15]; in the same studies these MICs compared favorably against the MICs of currently recommended antibiotics (rifampicin 1–2 mg/l, amikacin 1 mg/l, clarithromycin 1 mg/l). Ciprofloxacin has also been shown to have rapid bactericidal activity in humans against *M. tuberculosis* [24]–[26], and has been used to successfully treat other non-tuberculous mycobacterial infections including *M. marinum* [27], the species most closely related to *M. ulcerans*. Moxifloxacin similarly has good in vitro activity (MIC 0.25) [15]. In the mouse footpad model, moxifloxacin has bactericidal activity against *M. ulcerans* and, when combined with rifampicin, is as effective as combinations of rifampicin/streptomycin, rifampicin/amikacin and rifampicin/clarithromycin [8], [18]. Furthermore, other factors that favor the use of FQs include their high oral bioavailability (78%) [16] and excellent bone and tissue penetration [17]. Nevertheless, we would caution that fluoroquinolones should not be used as monotherapy for *M. ulcerans* treatment as there is the potential for the development of resistance, as has been shown to occur when FQs are used as monotherapy for *M. tuberculosis* [25]. Moxifloxacin may be favoured over ciprofloxacin due to slightly better published MICs against *M. ulcerans*, the evidence from the mouse footpad models of which there is no similar published data for ciprofloxacin, greater potential barrier to resistance if effects are similar to that in *M. tuberculosis* [25], and once-daily administration. A constraint at present is its significant increased cost compared to ciprofloxacin, with a cost of $671 compared with $32 Australian dollars for an 8-week treatment course at our institution.

In mouse footpad models, rifampicin has the greatest bactericidal activity against *M. ulcerans* [8], [18], and thus is assumed to be the most active and important antibiotic in vivo, though there are no human studies of rifampicin monotherapy to confirm this. It is possible that the second agent, including the FQs, act only as bacteriostatic agents preventing the emergence of rifampicin resistance. Therefore we recommend rifampicin as the first antibiotic choice, to be combined with another agent. Our data indicate that FQs are an appropriate combination choice, especially in cases where other antibiotics such as clarithromycin or streptomycin are not tolerated, accepted by patients, or accessible, or are contraindicated. FQs, greatest advantage may be their potential to be combined with other oral antibiotics such as rifampicin to provide completely orally administered regimens in endemic settings. This allows outpatient care to be provided close to patient homes and avoids daily intramuscular injections, potentially increasing patient willingness to present early for diagnosis and increase adherence to treatment. Furthermore, ciprofloxacin is generically produced, reducing its cost, and is readily available in many resource-limited settings.

Antibiotics were most commonly given for between 1 and 3 months (53%) in our cohort. A proportion of cases (21; 23%) had successful outcomes with less than 30 days of treatment, although 55% of cases given less than 2 weeks of antibiotics remained culture positive. One case remained positive after 36 days of antibiotics but still achieved cure, reinforcing findings from previous studies that a small proportion of cases may remain culture positive after 1–2 months of antibiotics but still achieve cure with at least 8 weeks of antibiotics [4], [5]. The proportion of patients receiving antibiotic treatment significantly increased from 2005 as it became apparent in patients treated at Barwon Health that antibiotics were associated with a reduction in disease recurrences and permitted more conservative surgery to be performed [6].

The efficacy of treatment is also determined by the tolerance of the antibiotic regimens. In our study there were no significant differences in the tolerability of the 3 main oral antibiotic choices of rifampicin, clarithromycin, and ciprofloxacin. In addition, the complications mainly involved gastrointestinal intolerance with no significant sequelae. This differs from the significant incidence of serious adverse events previously described for alternative antibiotics such as streptomycin [5], [28] and amikacin [6]. It is important to note that the complication rates were higher in our study compared to studies from African populations [4], [5], [7]. This is likely due to the older patient population in our cohort where these medications are less well tolerated, especially from a gastro-intestinal viewpoint, and there is more potential for drug interactions. Nevertheless it underlines the importance of having a number of oral antibiotic combinations available if first-line antimicrobials need to be substituted on account of intolerance.

Finally, paradoxical reactions occurred in 9% of antibiotic treated cases. To our knowledge this is the first published rate of paradoxical reactions in a cohort of patients treated for *M. ulcerans*. In our study the reactions occurred as early as 2 weeks and as late as 3 months after antibiotics were commenced. Recently, paradoxical reactions occurring after the cessation of antibiotics have been described [29]. It is important that paradoxical reactions are considered and recognized during treatment, and distinguished from treatment failures, to avoid unnecessary antibiotic regimens changes or further surgery [19].

There are a number of limitations to our study. First, it is an observational cohort and thus there is the potential for unknown confounders to have affected the results. Despite this possibility, the treatment outcome results are highly significant. Second, some of the infections acquired from the local endemic region occurred in visitors to the region and were not managed by our health service (Barwon Health) but in the health services where they live. Although this may have introduced a selection bias, we feel it is unlikely that this would have changed the findings of our study. Third, all cases underwent surgical excision and therefore the outcomes may not be applicable to cases where antibiotics alone are used. We advocate for further randomized studies using FQ-containing antibiotic regimens without curative surgery be performed to provide further information.

In summary, antibiotics in combination with surgery may significantly increase treatment success for *M. ulcerans* infections. In addition, antibiotic regimens containing oral FQs combined with rifampicin can provide an effective and safe treatment option and should be further studied in the treatment of *M. ulcerans*.

## Supporting Information

Checklist S1 **Strobe checklist**
(PDF)
